# A Noncontact Force Sensor Based on a Fiber Bragg Grating and Its Application for Corrosion Measurement

**DOI:** 10.3390/s130911476

**Published:** 2013-08-29

**Authors:** Clara J. Pacheco, Antonio C. Bruno

**Affiliations:** Departamento de Física, Pontifícia Universidade Católica do Rio de Janeiro, Rua Marquês de São Vicente 225, Rio de Janeiro, RJ 22451-900, Brazil; E-Mail: clajopac@gmail.com

**Keywords:** fiber Bragg grating, magnetic force sensor, photonic force sensor, internal corrosion

## Abstract

A simple noncontact force sensor based on an optical fiber Bragg grating attached to a small magnet has been proposed and built. The sensor measures the force between the magnet and any ferromagnetic material placed within a few millimeters of the sensor. Maintaining the sensor at a constant standoff distance, material loss due to corrosion increases the distance between the magnet and the corroded surface, which decreases the magnetic force. This will decrease the strain in the optical fiber shifting the reflected Bragg wavelength. The measured shift for the optical fiber used was 1.36 nm per Newton. Models were developed to optimize the magnet geometry for a specific sensor standoff distance and for particular corrosion pit depths. The sensor was able to detect corrosion pits on a fuel storage tank bottom with depths in the sub-millimeter range.

## 1. Introduction

In the last decade, significant progress has been made towards the development of fiber Bragg grating (FBG) sensors, which have the advantages of being immune to electromagnetic interference, easy to multiplex, remotely accessible, stable for long-term measurements, and easy to cable. When an FBG is embedded in or bonded on the appropriate host material, one is able to monitor changes in several physical parameters. Any strain in the fiber at the Bragg grating will cause a shift of the reflected Bragg wavelength, which can be precisely detected in a number of ways [1]. Several sensors have been built to measure temperature [2], humidity [3], pressure [4], vibration [5], deformation and displacement [6], electric current [7] and magnetic field [8–10]. Similar approaches have also been used to build sensors to measure mechanical force by bonding FBGs on cantilever structures [11–13]. FBG sensors have been found useful in the medical area [14–16] and in the construction industry, monitoring corrosion in reinforced steel bars [17,18].

More recently, FBG sensors have been used in the oil and gas industry to monitor corrosion in pipelines, offshore platforms, and fuel storage tanks [19]. Failures due to corrosion can be responsible for over 25% of the total failures on the oil and gas industry [20]. In order to ensure proper flow and storage of oil and gas, conforming to environmental laws, regulations and standards, the need for nondestructive techniques that provide reliable corrosion monitoring has never been higher.

We used a different approach that does not rely on the bonding of the Bragg grating to a specific substrate to develop a contactless force sensor; the sensor employs one FBG and a small permanent magnet attached to the tip of the optical fiber. Analytical and finite element models were developed to optimize and calibrate the sensor, and tests were performed with both fabricated and natural corrosion pits present in samples of a fuel storage tank bottom. The developed sensor can detect corrosion with sub-millimeter depths and has unique properties: it is passive with no need of local power sources, and can be used to monitor corrosion in remote sites and in places that are difficult to access.

## 2. Operational Principle

If a mechanical strain *ε* is induced in an FBG with Bragg wavelength *λ_B_*, the corresponding wavelength shift *Δλ_B_* can be written as:

(1) $$\Delta \lambda_{B}=(1-p_{e})\lambda_{B}ɛ$$

where *p_e_* is the effective strain-optic coefficient of the fiber. In the above expression, the temperature effect was omitted. It is possible to successfully compensate for this effect by adding a second FBG [12]. Expressing the mechanical strain in terms of force *F_m_*, cross-sectional area of the optical fiber *a* and its Young's modulus *E*, the wavelength shift can now be stated as:

(2) $$\Delta \lambda_{B}=(1-p_{e})\lambda_{B}\frac{F_{m}}{Ea}$$

**Figure 1.**
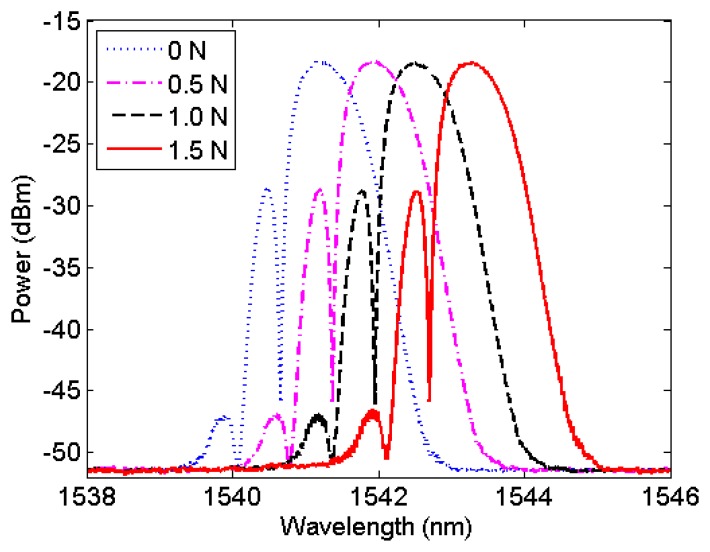
Power wavelength spectrum measured with the interrogation system while mechanically pulling an optical fiber with a Bragg grating. The force is monitored by an electronic dynamometer.

Figure 1 shows the optical power spectrum that was measured with a commercial FBG Interrogation System (Micron Optics sm 125–200, Atlanta, GA, USA). Side-lobe peaks appear because the Bragg grating used was not apodized [21]. It had an at-rest (dotted line) wavelength peak at 1,541.20 nm. Strains were induced into the FBG by fixing one end to a supporting structure and pulling the loose end by a dynamometer (Lutron Force Gauge FG-20 kg, Taipei, Taiwan) until the dynamometer reads 0.5 N, 1.0 N and 1.5 N. These strains increased the reflected wavelength peak to 1,541.88 nm (dash-dotted line), 1,542.55 nm (dashed line), and 1,543.24 (solid line) respectively. These simple measurements, after a linear fitting, indicate an FBG force sensitivity of 1.36 nm/N, which is in good agreement with the values reported in the literature for a standard, single-mode optical fiber [13,22].

**Figure 2.**
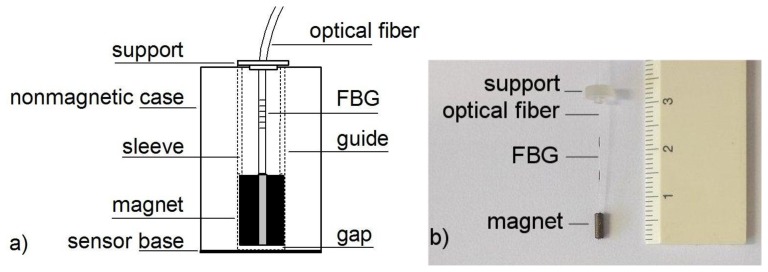
(**a**) Schematic drawing of the FBG magnetic force sensor; (**b**) Photograph of the sensor inside the nonmagnetic case, containing a 2.0 mm diameter magnet, 5.0 mm long and the optical fiber. The Bragg grating was inscribed approximately in the middle of the 2 dark marks on the optical fiber. The scale is in centimeters.

To turn the FBG into a contactless force sensor, we attached a small, cylindrical magnet that was magnetized in the direction of its axis to the loose end of the optical fiber by a tiny hole in the center; therefore, the tip of the optical fiber could be inserted into the hole in the magnet and firmly glued using a low-viscosity adhesive (Loctite 496, Henkel, São Paulo, Brazil). We used NdFeB and SmCo magnets (Logimag, Hong Kong, China) that were a few millimeters in diameter and a few millimeters long. These small magnets are routinely employed in optical isolators and as rotors for quartz watches. As observed in the schematic in Figure 2a, we encapsulated the FBG and magnet in a nonmagnetic Teflon cylinder with a gliding cavity. The Teflon cylinder serves three purposes: to protect the Bragg grating, to minimize friction, and to keep unwanted ferromagnetic parts away from the magnet. A plastic sleeve eliminates the potential upper motion of the magnet and FBG so no shear, compression, and bending can affect the sensor device. We glued the upper part of the optical fiber to a support using the same low-viscosity adhesive.

## 3. Analysis of the Magnet Geometry

The role of the spatial dependence of the magnetic field, caused by the magnet, to the applied force can be explicitly determined if we calculate the force on a ferromagnetic body with the principle of virtual work. The principle of virtual work states that the force on a body can be calculated by taking the gradient of the energy change, which is caused by its virtual displacement, in the volume occupied by the body [23]. Suppose a ferromagnetic body has a volume *v*, the magnetic force *F_m_* is given by:

(3) $$F_{m}=\nabla \int_{v}(u_{B}-u_{B0})dv$$

where *u_B_* is the magnetic energy density in the volume occupied by the ferromagnetic body and *u_B0_* is the magnetic energy density in the same volume in the absence of the body. Using *μ_0_* as the permeability of free space, *χ* as the body susceptibility, *D* as an effective demagnetization factor of the body [24], and *H* as the magnetic field, the magnetic force *F_m_* is given by:

(4) $$F_{m}=\frac{1}{2}\mu_{0}\left(\frac{\chi -D\chi }{1+D\chi }\right)\int_{v}\nabla H^{2}dv$$

Assuming a magnetic field gradient in only one direction, e.g., in the z direction:

(5) $$F_{m}=\mu_{0}\left(\frac{\chi -D\chi }{1+D\chi }\right)\int_{v}H\frac{dH}{dz}dv$$

Thus, the force caused by the magnet in a ferromagnetic body is a function of the body susceptibility, demagnetization factor and the product of the magnetic field and the magnetic field gradient integrated over the volume of the body. Considering the objective of assessing the influence of the magnet geometry in the proposed sensor, it will be useful to analyze product of the magnetic field and the magnetic field gradient generated by the magnet as a function of the radius and length of the magnet. The axial magnetic field outside a cylindrical magnet, magnetized in the direction of its axis, of radius *R* and length *L*, as a function of the axial distance *z* from the magnet face, can be given by [25]:

(6) $$H_{z}=\frac{M_{0}}{2}\left[\frac{L+z}{\sqrt{R^{2}+{\left(L+z\right)}^{2}}}-\frac{z}{\sqrt{R^{2}+z^{2}}}\right]$$

where *M_0_* is the magnetization of the permanent magnet. Differentiating Equation (6) with respect to *z* yields:

(7) $$\frac{dH_{z}}{dz}=\frac{M_{0}}{2}\left\{\frac{1}{{\left[R^{2}+{\left(L+z\right)}^{2}\right]}^{\frac{1}{2}}}-\frac{{\left(L+z\right)}^{2}}{{\left[R^{2}+{\left(L+z\right)}^{2}\right]}^{\frac{3}{2}}}-\frac{1}{{\left[R^{2}+z^{2}\right]}^{\frac{1}{2}}}-\frac{Z^{2}}{{\left[R^{2}+z^{2}\right]}^{\frac{3}{2}}}\right\}$$

**Figure 3.**
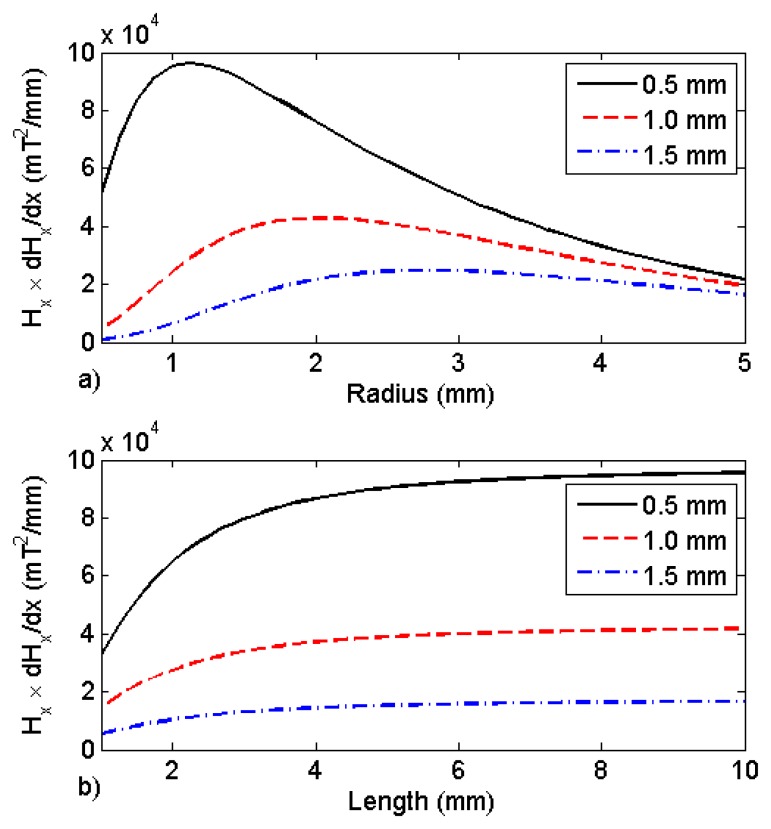
Product of the theoretical magnetic field with the magnetic field gradient (**a**) *versus* the radius of the magnet and (**b**) *versus* its length, for three different standoff distances (0.5 mm, 1.0 mm, and 1.5 mm).

Both expressions are approximations because they do not take into account the hole in the magnet center. Nevertheless, the product of the two expressions will give rough estimates of the dimensions of the magnet radius and length that will maximize the magnetic force at a specific distance. Figure 3a shows the product of the magnetic field with magnetic gradient *versus* the magnet radius when the magnet is placed at three different distances (0.5 mm, 1.0 mm, and 1.5 mm) from the ferromagnetic body. The magnet length used was 5.0 mm and *M_0_* = 800 kA/m. Figure 3b shows the product of the magnetic field and magnetic gradient *versus* the magnet length. The magnet radius used was 1.5 mm. The calculations were made at the same three distances. It can be noted that the magnet radius plays an important role in the product of the magnetic field and the gradient amplitudes, especially at small distances from the magnet. Note that there is only a small influence in the product for lengths larger than 5.0 mm. When the magnet length is 5.0 mm, it is already equal to 95% of the maximum value of the product. A practical constraint concerning the magnet size is related to its weight. For example, the mass of a magnet with 1.5 mm radius, 5.0 mm long is 0.3 g. This corresponds to a weight of about 3 mN. In order to neglect the gravity effect, the smallest magnetic force between the sensor and the ferromagnetic component must be much larger than that. This will limit the maximum distance the sensor will be able to measure with that specific magnet. Another practical limitation is the maximum tensile strength of the optical fiber used. A theoretical maximum force of about 13 N was obtained by using Equation (2), a maximum strain equal to 1.2% and typical optical fiber parameters, *E* = 90 GPa and *a* = 1.23 × 10^−8^ m^2^ (125 μm optical fiber diameter). At the laboratory, we were able to apply up to 7.4 N (Δλ = 10 nm) to the optical fiber without breaking it. This limit will have to be taken into account when choosing the sensor magnet and standoff distance.

## 4. Sensor Calibration

**Figure 4.**
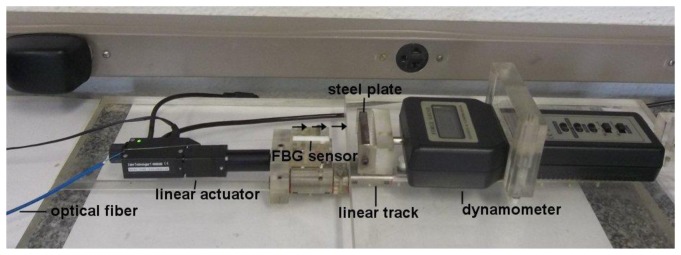
Photograph of the experimental setup for sensor calibration. The linear actuator displaces the FBG sensor towards the steel plate as shown by the arrows. The interrogation systems measure the wavelength change and the dynamometer measures the attractive force between the sensor magnet and the steel plate. The FBG sensor Teflon case has 6.0 mm diameter and it is 3.0 cm long.

Based on the previous analysis, we built FBG sensors using magnets that were 5.0 mm long with radii equal to 1.5 mm and 1.0 mm (sensors A and B, respectively). We also tested magnets that were 10 mm long with radii equal to 1.5 mm and 1.0 mm (sensors C and D, respectively). The FBG sensor was calibrated in terms of force by sliding it towards a 6.7 mm thick, 40 mm × 20 mm low-carbon steel plate while measuring the attractive force with an attached dynamometer. The measurements were performed on a linear track with a linear actuator with 0.1 μm resolution (Zaber T-NA08A50, Vancouver, Canada), as shown in Figure 4.

**Figure 5.**
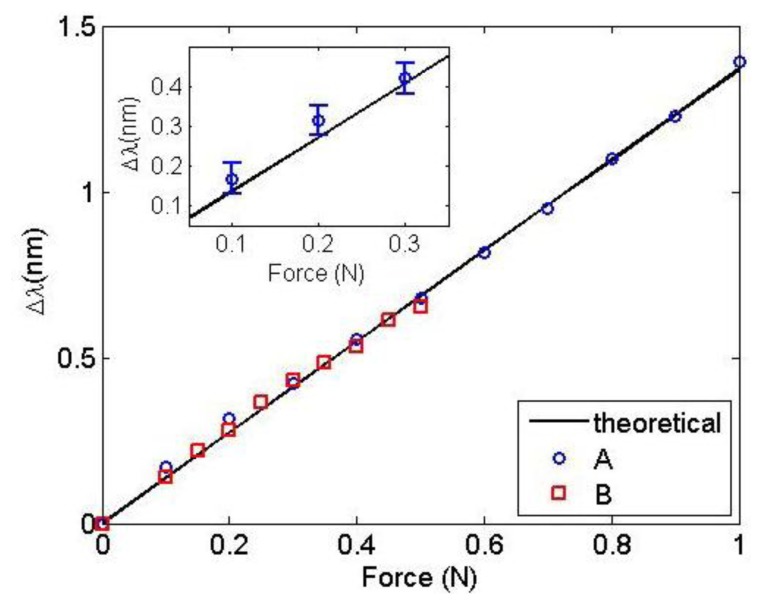
Bragg wavelength shift as a function of the force between sensors A and B and the ferromagnetic plate. The solid line was obtained from Equation (2). The inset shows a detail of sensor A measurements with error bars.

Figure 5 shows the result of the Bragg wavelength shift *versus* the magnetic force between sensors A and B and the steel plate. Sensor A was evaluated from 0 N to 1.0 N (circles) and sensor B from 0 N to 0.5 N (squares). The same figure also shows the theoretical Bragg wavelength shift using Equation (2) with *λ_B_* = 1,541.20 nm, a = 1.23 × 10^−8^ m^2^, *p_e_* = 0.22 and *E* = 71.5 GPa (solid line). The Young's Modulus value was obtained from a least-squares fitting routine and is in very good agreement with the values reported in the literature [17].

The average uncertainty obtained in the experimental values and Equation (2) was approximately 0.020 nm for both sensors. Thus, according to Figure 5, the experimental sensitivity of the FBG magnet force sensor is 1.36 ± 0.02 nm/N. This value is the same one achieved by mechanically pulling the optical fiber, as shown in Figure 1. This result demonstrates that the constructed sensors measure force without contact with the best possible sensitivity for a non-tapered optical fiber [22]. Additionally, the inset of Figure 5 shows the first three points measured with sensor A and their respective error bars. The departure from the linear behavior can be attributed to the overall noise present in our system, which may originate from the FBG interrogation system electronics, vibrations in our experimental setup and/or friction between the magnet and the nonmagnetic case.

## 5. Sensor Application for Corrosion Measurement

### 5.1. Wavelength Change versus Distance

**Figure 6.**
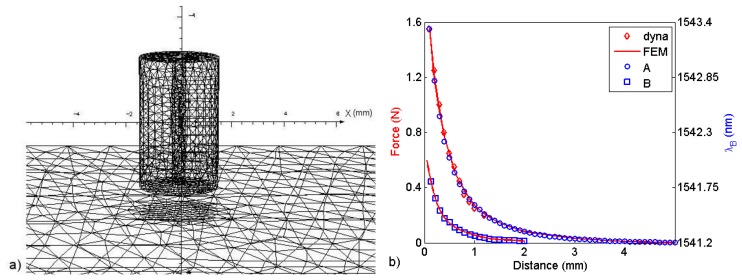
(**a**) Three-dimensional FEM model; (**b**) FEM results for the force *versus* distance from the steel plate (solid line). The dynamometer measurements (diamonds) and the measured wavelengths of sensor A (circles) and sensor B (squares) are also shown.

In order to do quantitative measurements, an additional calibration was performed with a three dimensional model using finite elements (FEM) with approximately 177,000 elements (Opera-3D, Cobham, Dorset, United Kingdom). We modeled the magnets in sensors A and B with Br = 1.1 T and Hc = 0.82 MA/m and the steel plate (20 mm × 40 mm × 6.7 mm) with a nonlinear BH curve that was determined experimentally. The modeled magnets had a 0.5 mm-diameter pass-through hole. The model geometry and the simulation results for sensors A and B are shown in Figure 6, respectively, where the Bragg wavelength and dynamometer measurements *versus* distance are also shown. Note that the maximum force measured with sensor A, at 100 μm from the ferromagnetic plate, was about 1.6 N, well below the maximum tensile strength of the optical fiber mentioned at the end of Section 3.

### 5.2. Validation Based on Controlled Fabricated Corrosion Pits

**Figure 7.**
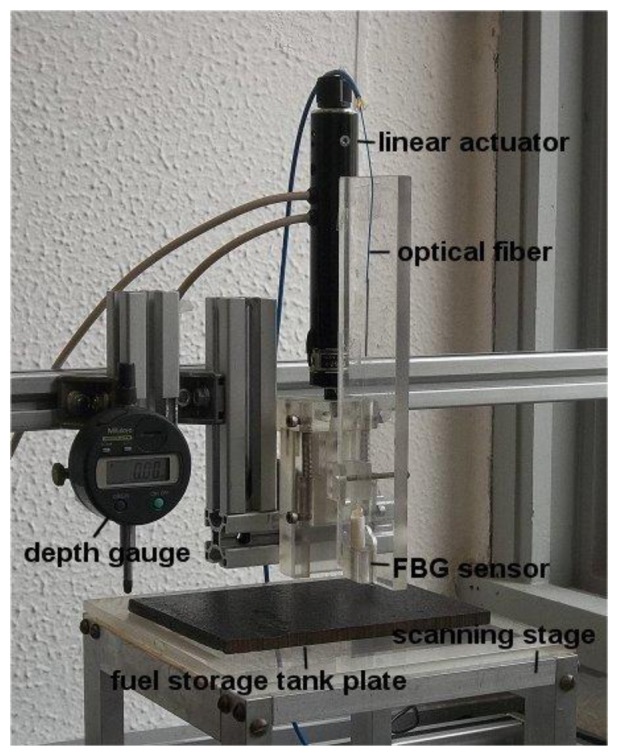
XYZ-motorized stage used to scan steel plates with fabricated semi-spherical and natural corrosion pits under the depth gauge and FBG force sensors.

To test the sensors on steel samples with corrosion pits, we used the experimental setup shown in Figure 7. The setup consists of an XYZ-motorized stage containing a depth gauge (Mitutoyo 543–682B, São Paulo, Brazil) and the FBG force sensor. We positioned steel plates (150 mm × 150 mm × 6.7 mm) containing fabricated semi-spherical corrosion pits to scan them under the FBG sensor and depth gauge.

**Figure 8.**
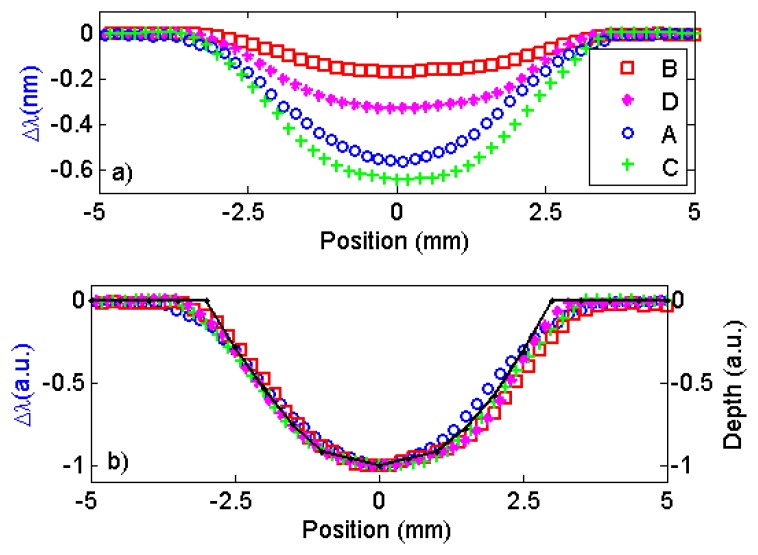
Change in the Bragg wavelength as the plate containing a fabricated semi-spherical corrosion pit is scanned under the FBG sensors. (**a**) Sensor A has a magnet 5.0 mm long with radius 1.0 mm (squares). Sensor B has a magnet 5.0 mm long with radius 1.5 mm (circles). Sensor C has a magnet 10 mm long with radius 1.0 mm (diamonds). Sensor D has a magnet 10 mm long with radius of 1.5 mm (crosses); (**b**) Normalized wavelength change and depth profile (solid line) *versus* distance. The scans were performed on a line passing over the center of the pit.

We started by scanning a fabricated, semi-spherical pit that was 6.0 mm in diameter and 0.5 mm deep under four sensors that were built with different magnet geometries (sensors A, B, C, and D). The results can be observed in Figure 8a, where the changes in the Bragg wavelength Δλ for the four sensors are plotted *versus* the position on a line passing over the center of the pit. In this measurement, the sensor standoff distance was 0.5 mm from the plate. As observed, all of the sensors were able to detect the 0.5 mm-depth pit and, as expected, the sensor with the larger magnet generated the larger change in the reflected wavelength. Notice that, in Figure 8b, normalizing the wavelength changes and the depth profile measured with the depth gauge produces a very good match, indicating that the wavelength change obtained by all sensors accurately describes the depth profile of the corrosion pit.

**Figure 9.**
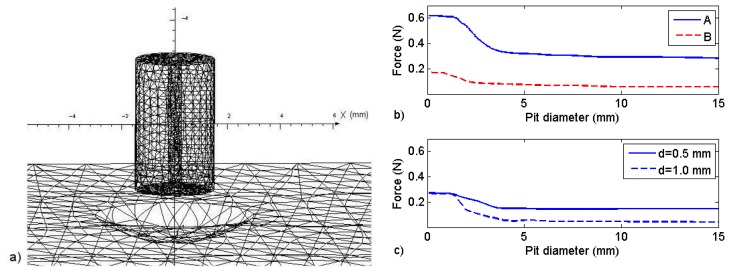
(**a**) Three-dimensional FEM model including a magnet with a 1.5-mm radius and a 6.0-mm diameter semi-spherical pit; (**b**) FEM results of the force for sensor A (solid line) and sensor B (dashed line) with a 0.5-mm standoff distance *versus* the pit diameter with a 0.5-mm depth; (**c**) The force on sensor A with a 1.0-mm standoff distance *versus* the pit diameter having 0.5-mm depth (solid line) and 1.0-mm depth (dashed line).

To use Figure 6b to properly determine the depth of a corrosion pit, we used the FEM model to study the effect of the corrosion pit border on the force (wavelength change) measured by the FBG sensor. This effect was accomplished by modeling semi-spherical pits in the steel plate, as shown in Figure 9a.

It is expected that if the pit diameter is less than the diameter of the sensor magnet, the measurement will be influenced by the pit border; therefore, the decrease in the magnetic force will be less evident when the sensor is over the pit. This result was obtained in the simulations for the force on the magnet as a function of the corrosion pit diameter. Figure 9b shows how the force on sensors A and B changes as a function of the pit diameter when the sensors are placed over the center of a corrosion pit 0.5 mm deep. The standoff distance of both sensors in relation to the plate was 0.5 mm. The effect of the pit border effect was evaluated with the increase of the magnetic force produced, compared to the force that would be produced if the pit had a much larger (infinite) border, as observed in Figure 6b. For sensor A, the effect of the border is less than 10% when the pit diameter is approximately twice the size of the magnet (solid line), and there is similar behavior for sensor B (dashed line). Figure 9c shows the effect of the pit border on sensor A when the standoff distance is increased to 1.0 mm. The effect becomes less evident at larger standoff distances, as the change in the force is less than 10% when the diameter of the pit is approximately 30% larger than the diameter of the sensor (solid line). However, when the depth is increased to 1.0 mm (dashed line) but the pit size remains the same, the border effect increases to 70%. The change in the force returns to less than 10% when the pit diameter is again approximately twice the diameter of the sensor magnet. Thus, once the change in wavelength is measured, to use Figure 6b to assess the pit depth, one must be sure that the pit diameter is at least twice the diameter of the FBG magnet.

**Figure 10.**
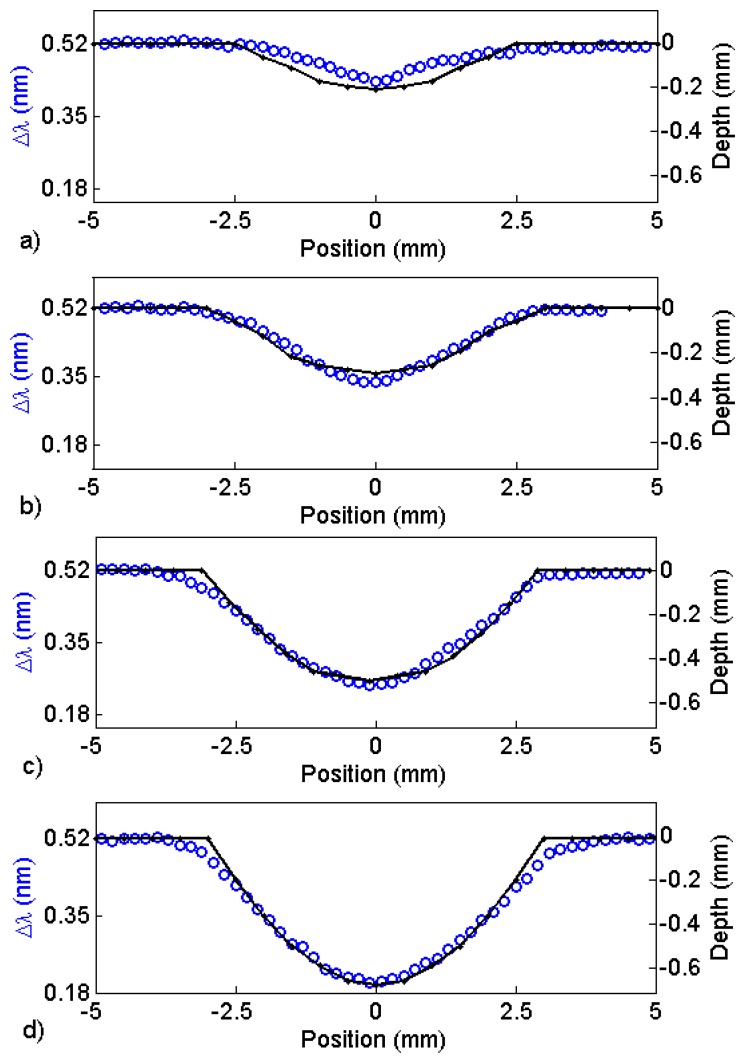
The changes in the wavelength (circles) detected by sensor A that are obtained when fabricated, semi-spherical pits with 6.0 mm diameter and four different depths (**a**) 0.17 mm; (**b**) 0.3 mm; (**c**) 0.5 mm and (**d**) 0.7 mm are scanned under the sensor with an 0.8-mm plate standoff distance. The depth profiles measured by a depth gauge are also shown (solid line).

**Figure 11.**
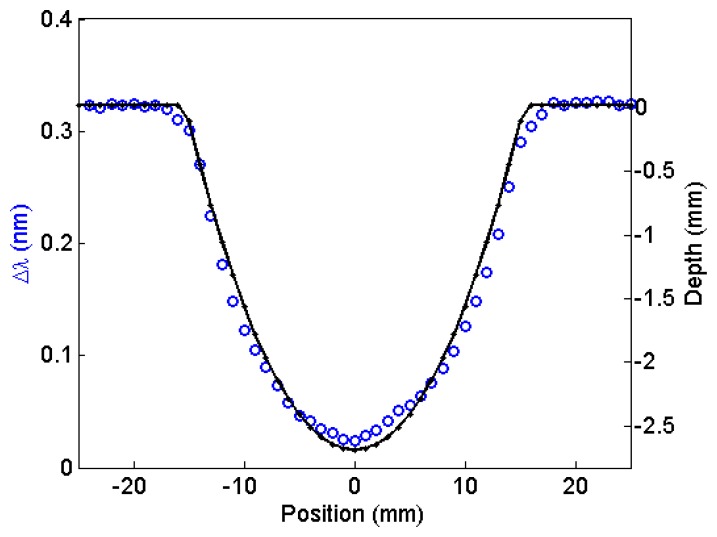
Change in wavelength detected by sensor A (circles) obtained when a fabricated semi-spherical pit with 30-mm outside diameter and 2.7-mm depth is scanned under sensor A at a 1.2-mm standoff distance. The depth profile measured by a depth gauge is also shown (solid line).

Figure 10 shows changes in wavelength (circles) when scanning sensor A over fabricated, semi-spherical pits with 6.0 mm diameter and four different depths 0.17 mm; 0.3 mm; 0.5 mm and 0.7 mm. The standoff distance was 0.8 mm. The depth profiles measured by a depth gauge are shown as well (solid line). A larger and deeper semi-spherical fabricated pit was also measured using sensor A with the standoff distance increased to 1.2 mm. The pit diameter was 30 mm, and it was 2.7 mm deep. The resulting measurement is shown in Figure 11. The change in wavelength (circles) was related to the pit depth (solid line) by using Figure 6b. The value obtained for the depth was 2.6 mm. Notice that at this standoff distance; we are at the full dynamic range of sensor A.

### 5.3. Testing with Real Corrosion Sample

**Figure 12.**
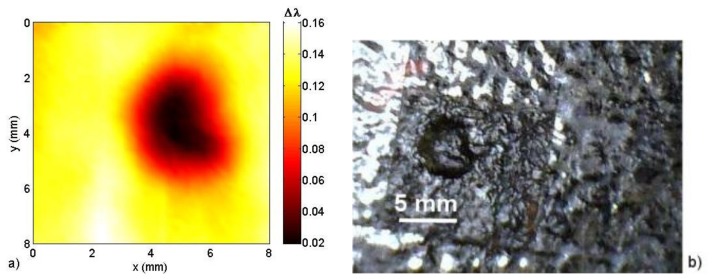

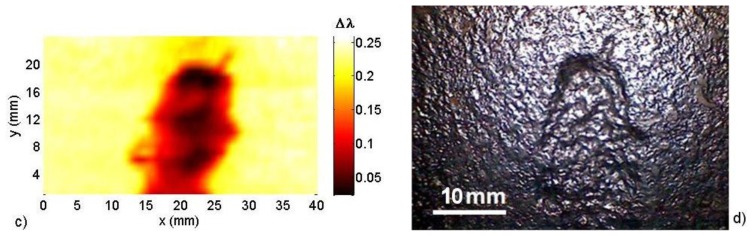
(**a**) Image obtained by scanning a natural corrosion pit under sensor B; (**b**) The natural corrosion pit is 0.4 mm deep; (**c**) Image obtained by scanning an extended corrosion area under sensor B; (**d**) Extended corrosion area. The largest depth in the area is 0.7 mm. The images have been displayed to present maximum dynamic range.

The smaller sensor B was used to detect a natural corrosion pit that was found in the bottom plate of a fuel storage tank, as shown in Figure 12b. The natural pit is 0.4-mm deep and approximately 5.0 mm in diameter. Scanning the pit under the FBG sensor at a 0.5-mm standoff distance, we obtain the image that can be observed in Figure 12a. Additionally, an extended corrosion area found on the same plate, shown in Figure 12d, was also detected by sensor B. The image obtained can be observed in Figure 12c at a 0.3-mm standoff distance. The maximum depth was 0.7 mm, which corresponds to a change in *Δλ* from 0.23 nm to 0.04 nm.

## 6. Conclusions

A simple fiber Bragg grating, magnetic force sensor was proposed and built. The sensor can be used to monitor the internal corrosion of ferromagnetic structures without being attached to the structure. A theoretical analysis of the magnetic field caused by the sensor magnet was performed, and sensors were designed to maximize the magnetic force for standoff distances in the millimeter and sub-millimeter range. A sensitivity of approximately 1.36 ± 0.02 nm/N was obtained, which is the same sensitivity that is obtained when the FBG is pulled mechanically. Calibration curves relating the force (change in wavelength) and distance from the sensor to the ferromagnetic material were obtained using finite element models. Additionally, when using the models, we found that the size of the pit border must be at least twice the diameter of the sensor magnet to use the calibration curves. Taking this constraint into account, the calibration curves were successfully used in experimental measurements to detect corrosion pits with depths in the sub-millimeter range. We did not observe any important hysteresis effects on the FBG sensors, as the detected wavelength change was about the same at both sides of the corrosion pits.
